# Necrological

**Published:** 1880-04

**Authors:** 


### NECROLOGICAL.

" Death is the crown of life :
Were death denied, poor man would live in vain."

At the house of his son-in-law, H. W. Merchant, of Worcester, N. Y., on Sunday, February 11, 1880, of apoplexy, Dr. Tunis Cooper, aged 88 years.

In Philadelphia, suddenly, on the 3d instant, George F. Goersen, M. D., in the 63d year of his age.

Dr. A. B. Haile, a physician of about forty years' practice in Norwich, Ct., died March 9th, at the age of 74 years.

At his late residence, No. 63 Madison Avenue, New York, on March 4th, J. A. Ward, M. D.

Dr. D. G. Thomas, a leading physician of Central New York, died at Utica on Saturday, aged 74.

Dr. J. H. Darrell, a well-known Washington dentist, died suddenly on Saturday of congestion of the brain.

We sincerely regret to learn the death of the wife of our old and valued friend, Prof. G. Troup Maxwell, M. D., of Wilmington, Del. The sad event occurred on March 21, 1880, at her residence in that city. A friendship that has known no abatement for nearly a quarter of a century as co-professors and associates, through prosperity and in adversity, through war and in peace, causes us to sympathize most keenly in anything that might affect him upon whom this dispensation has fallen. And we most heartily commend him and his bereaved ones to the tender mercy of Him who tempers the wind to the shorn lambs.

